# Urinary Biomarkers in Parkinson’s Disease: A Structured Integrative Review of Pathophysiological Pathways

**DOI:** 10.3390/medsci14020258

**Published:** 2026-05-17

**Authors:** Halyne Queiroz Pantaleão Santos, Nairo Massakazu Sumita, Carlos Alberto-Silva, Marcela Bermudez Echeverry

**Affiliations:** 1 Natural and Humanities Sciences Center (CCNH), Experimental Morphophysiology Laboratory, Federal University of ABC (UFABC), São Bernardo do Campo 09606-070, SP, Brazil; halyne.queiroz@ufabc.edu.br; 2 Central Laboratory Division, Hospital das Clínicas da Faculdade de Medicina, Universidade de São Paulo (HCFMUSP), LIM-03, São Paulo 05403-010, SP, Brazil; nairo.sumita@hc.fm.usp.br; 3 Mathematics, Computation and Cognition Center (CMCC), Federal University of ABC (UFABC), São Bernardo do Campo 09606-070, SP, Brazil

**Keywords:** neurodegenerative disorders, proteomics, oxidative stress, mitochondrial dysfunction

## Abstract

Background/Objectives: Parkinson’s disease (PD) is a progressive neurodegenerative disorder characterized by complex and interconnected pathophysiological mechanisms, including mitochondrial dysfunction, oxidative stress, neuroinflammation, lysosomal impairment, and altered neurotransmitter metabolism. Unlike cerebrospinal fluid or blood, urine offers a truly non-invasive source of biomarkers, reflecting systemic metabolic changes and renal protein excretion linked to neurodegeneration. This review aims to critically synthesize current evidence on urinary biomarkers in PD and to organize this heterogeneous literature into pathophysiologically meaningful domains. Methods: A comprehensive literature search of human studies investigating urinary biomarkers in PD was performed. Eligible studies were comprehensively analyzed and classified according to dominant biological pathways. To facilitate interpretation, findings were organized into six thematic domains: genetic and protein-based biomarkers; metabolic pathways and mitochondrial dysfunction; oxidative stress and neuroinflammation; gut–brain-axis-related metabolites; hormonal and systemic biomarkers; and emerging exploratory markers. Results were summarized in domain-specific tables and integrated using a conceptual framework. Results: A total of 32 human studies met the inclusion criteria, revealing diverse urinary molecular signatures associated with PD across multiple biological domains. Genetic and protein-based markers, including LRRK2-related proteins, α-synuclein species, and lysosomal lipids, showed potential for disease stratification. Metabolomic studies consistently identified alterations in acylcarnitines, organic acids, and amino acid metabolism, reflecting mitochondrial dysfunction. Biomarkers related to oxidative stress, immune activation, gut microbiota metabolism, and hormonal regulation further highlighted the systemic nature of PD. However, most individual biomarkers lacked disease specificity and exhibited methodological heterogeneity. Conclusions: Current evidence supports urine as a valuable source of systemic biomarkers reflecting multiple pathophysiological processes in PD. While single urinary markers remain insufficient for clinical application, integrated omics-based approaches—particularly metabolomics and peptidomics/proteomics—hold promise for identifying combinatorial biomarker signatures. Future longitudinal and standardized studies are required to enhance specificity and translational potential for non-invasive diagnosis and disease monitoring in PD.

## 1. Introduction

Parkinson’s disease (PD) is a progressive neurodegenerative disorder with a high prevalence among middle-aged and older individuals, characterized by asymmetrical resting tremor, rigidity, hypokinesia, and postural instability as its cardinal motor symptom [1], which results from the progressive degeneration of midbrain dopaminergic neurons in the substantia nigra pars compacta [2,3]. In 2021, approximately 11.77 million people worldwide were affected by Parkinson’s disease (PD). PD prevalence is highly age-dependent, affecting approximately 1% of the population over 60 years of age and rising to nearly 4% in individuals aged 80 or older. With the global aging population, these numbers are projected to double by 2040, highlighting the urgency for accessible biomarkers [4]. Furthermore, disability-adjusted life years (DALYs) attributable to PD have increased by approximately 81% since 2000, while disease-related mortality has increased by more than 100%. Moreover, disability-adjusted life years (DALYs) attributable to PD have increased by approximately 81% since 2000, while disease-related mortality has risen by more than 100% [5].

Although mutations in the Leucine Rich Repeat Kinase 2 (LRRK2) and Glycocerebrosidase (GBA) genes (the latter encoding the lysosomal enzyme glucocerebrosidase, GCase) represent a potential link between genetic and idiopathic forms of PD [6,7], the SNCA gene, which encodes α-synuclein, remains the most extensively studied, as its protein product is a major component of proteinaceous inclusions known as Lewy bodies (LBs) and Lewy neurites, both considered pathognomonic neuropathological hallmarks of PD [8]. Indeed, genetic variation in SNCA—particularly common polymorphisms—has been consistently associated with an increased risk of developing PD, with several studies also suggesting an earlier age at disease onset [8]. Recent evidence indicates that the PD Biomarker Program employing α-synuclein (αSyn) seeding amplification assays (αSyn-SAAs) provides improved accuracy in distinguishing individuals with PD from healthy controls [9], thereby enabling earlier disease staging and monitoring of progression. Nonetheless, important limitations remain, including reduced specificity for differentiating PD from other synucleinopathies, variable assay performance in patients with genetic forms of PD (particularly those carrying LRRK2 or SNCA variants), heterogeneity in nigrostriatal neurodegeneration with or without Lewy body pathology at autopsy, and the presence of α-synuclein in multiple biochemical states, including soluble, aggregated, and post-translationally modified forms [10]. In addition, patients with advanced motor impairment often require special clinical care when undergoing lumbar puncture for the detection of misfolded α-synuclein in cerebrospinal fluid (CSF), and it remains unclear how reliably αSyn-SAAs can identify PD at pre-motor stages or accurately reflect disease severity and progression [11].

Like other neurodegenerative disorders, PD arises from a complex interplay of genetic, epigenetic, and environmental factors. One of the major challenges in PD research is the identification of reliable biomarkers that exhibit a meaningful association with clinical stages, enable prediction of disease progression, and allow monitoring of therapeutic efficacy [12]. Accordingly, biomarkers in PD can be broadly classified into four main categories: clinical, imaging, biochemical, and genetic. Biochemical and neurochemical biomarkers include dopamine-related metabolites, amino acids, and other small molecules detected in biological fluids such as blood, serum, and CSF, as well as microRNA-based analyses and metabolomics, which represent advanced approaches for comprehensive metabolic profiling of body fluids [12].

Considering all the factors mentioned above, and given accumulating evidence that mitochondrial dysfunction, oxidative stress, lysosomal dysregulation, and impairment of the ubiquitin–proteasome system contribute to the progressive loss of dopaminergic neurons in the substantia nigra pars compacta in PD [13,14], there is a clear need for simpler and less invasive approaches to investigate molecular biomarkers [15]. Periodic assessment of such biomarkers may facilitate the establishment of early correlations with PD risk factors, enable the detection of subtle brain-related pathological changes in individuals who are still asymptomatic, and support the evaluation of whether therapeutic interventions are effectively slowing or halting disease progression.

In this context, urine represents an abundant, easily accessible, and non-invasive biological sample. It reflects systemic physiological and pathological changes and contains not only proteins derived from the kidneys and urinary tract but also filtered plasma proteins originating from distant organs, including the brain [16,17]. The focus on urinary biomarkers is justified by the clinical need for longitudinal monitoring tools that are cost-effective and patient-friendly. Furthermore, the presence of brain-derived extracellular vesicles and altered metabolites in urine provides a unique biochemical window into the pathophysiological pathways of Parkinson’s Disease, bypassing the risks associated with invasive sampling [16,17]. Accordingly, this review summarizes and critically discusses recent advances in the identification and development of urinary biomarkers for PD, highlighting their potential applications in disease diagnosis and monitoring.

Despite the growing body of literature on biomarkers for PD, most reviews have focused on cerebrospinal fluid, blood-based markers, or neuroimaging approaches, while urinary biomarkers remain comparatively underexplored. Moreover, existing studies often address isolated molecular classes without integrating genetic, proteomic, metabolomic, and extracellular vesicle-associated findings into a unified framework. In this context, a comprehensive and updated synthesis of human studies investigating urinary biomarkers in PD is warranted. This review employs a structured approach to compile and critically analyze current evidence on urinary biomarkers associated with PD, emphasizing their pathophysiological relevance, methodological heterogeneity, and potential clinical applicability.

## 2. Methods

### 2.1. Study Design

This study is characterized as an integrative and structured narrative review. Unlike a systematic review, which aims strictly to answer a specific clinical question through restricted parameters, this integrative approach allows the inclusion of diverse methodologies and the synthesis of complex pathophysiological pathways, providing a more holistic theoretical framework for urinary biomarkers in PD. The objective of this study is to identify and synthesize the current evidence on urinary biomarkers associated with Parkinson’s disease. Given the exploratory nature of the available literature and the substantial heterogeneity across study designs, biomarkers, and analytical platforms, a formal review protocol was not prospectively registered. Nevertheless, the eligibility criteria, search strategy, and analytical framework were defined a priori and applied consistently throughout the review process, following selected principles of the PRISMA 2020 guidelines to enhance transparency and reproducibility. Considering this context and the lack of standardized and comparable reporting across studies, this review does not include a formal risk-of-bias assessment, certainty of evidence evaluation, or quantitative synthesis. Instead, the focus was placed on a qualitative and integrative analysis of the available evidence.

### 2.2. Sources of Information and Search Strategy

A comprehensive literature search was performed across the following electronic databases: PubMed, Embase, Scopus, Web of Science, Cochrane Library, Lilacs, Epistemonikos, Google Scholar, and the CAPES Portal. The search covered studies published from database inception up to the most recent available date at the time of the search, 9 April 2025. The search strategy combined controlled vocabulary (when available) and free-text terms related to PD and urinary biomarkers. Key search terms included combinations of: “Parkinson’s disease”, “urine”, “urinary biomarkers”, “metabolomics”, “proteomics”, “extracellular vesicles”, and “exosomes”, using Boolean operators (AND, OR). Reference lists of included articles were also manually screened to identify additional relevant studies.

### 2.3. Eligibility Criteria

Studies were selected according to predefined inclusion and exclusion criteria. Inclusion criteria were: (i) original studies conducted in human participants;(ii) studies including individuals with a clinical diagnosis of PD at any disease stage; (iii) evaluation of biomarkers measured exclusively in urine; (iv) studies reporting molecular, biochemical, proteomic, metabolomic, genetic, or extracellular vesicle-associated biomarkers. Exclusion criteria were: (i) studies conducted in animal models or cell cultures; (ii) studies assessing multiple biological fluids without isolated urinary data; (iii) reviews, meta-analyses, editorials, letters, conference abstracts, or case reports; (iv) studies with insufficient methodological information or unavailable full text.

### 2.4. Study Selection Process

**Figure 1:**
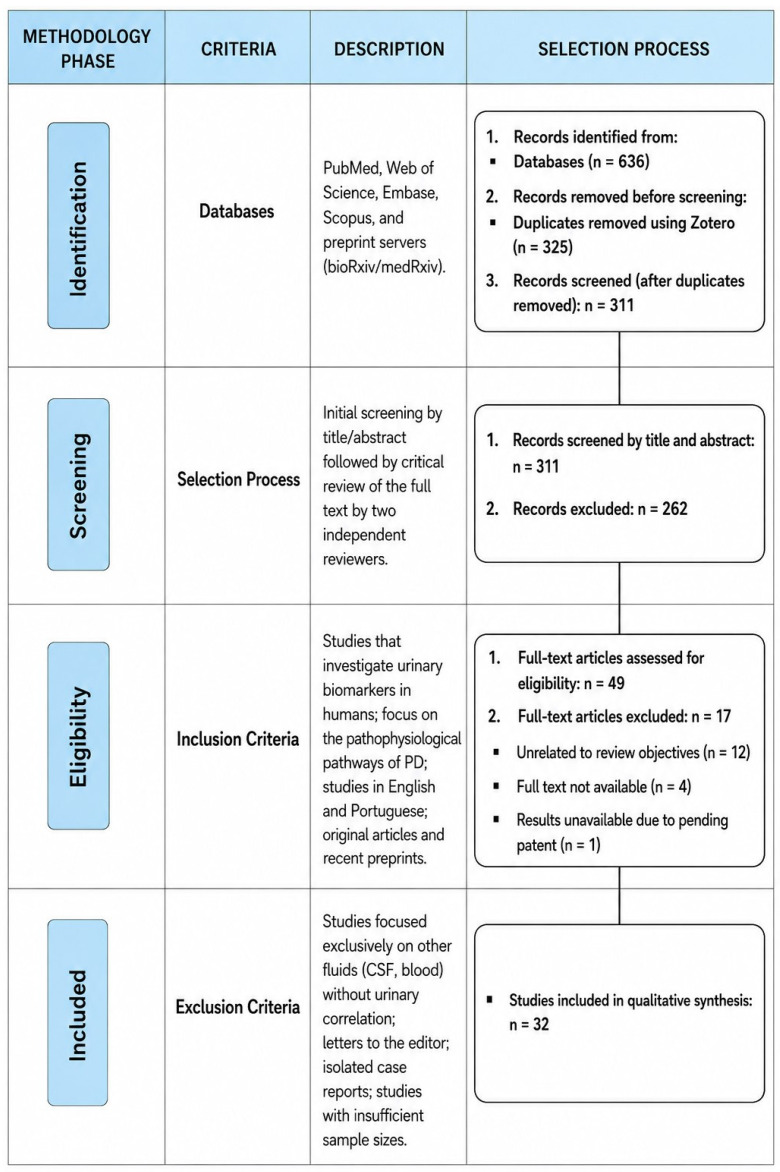
Flow diagram of study identification and selection, adapted from PRISMA 2020 recommendations, illustrating the identification, screening, eligibility, and inclusion of studies investigating urinary biomarkers in Parkinson’s disease.

All retrieved records were imported into Zotero (v9.0.3, Corporation for Digital Scholarship, Vienna, VA, EUA) reference management software, where duplicate entries were identified and removed. The remaining studies underwent an initial screening based on titles and abstracts to assess relevance. Subsequently, potentially eligible articles were evaluated through full-text review. The study selection process resulted in 636 records initially identified, of which 311 remained after duplicate removal. Following title and abstract screening, 49 articles were selected for full-text assessment. After exclusion of studies that did not meet the eligibility criteria or were unavailable in full, 32 studies were included in the final qualitative synthesis. The selection process is summarized in a PRISMA flow diagram (Figure 1).

In order to encompass the most recent scientific advancements, pre-prints were included in this review. We acknowledge that these documents have not yet undergone formal peer review, which may imply a potential instability in their reported results. However, their inclusion was deemed essential to reflect the current frontier of biomarker research, and they were critically appraised alongside peer-reviewed literature.

### 2.5. Data Extraction

Data extraction was performed using a standardized approach. The following information was collected from each included study: author(s), year of publication, study design, population characteristics, PD stage when available, urinary biomarkers investigated, analytical methodology, and main findings. Extracted data were organized into thematic categories according to the biological nature and pathophysiological relevance of the identified biomarkers.

### 2.6. Quality Assessment and Methodological Considerations

Due to the substantial heterogeneity among the included studies in terms of design, sample size, analytical platforms, and outcome measures, a formal quantitative risk-of-bias assessment was not performed. This decision was based on the lack of standardized reporting across studies and the absence of consistently comparable methodological parameters, which would limit the validity and interpretability of a structured bias assessment. Instead of applying a formal scoring system, methodological limitations were critically appraised through a qualitative approach, considering factors such as study design, sample size, analytical techniques, and reproducibility of findings. These aspects were discussed throughout the manuscript in the context of each biomarker category. In addition, no formal assessment of certainty of evidence was conducted, as the available studies did not provide sufficient consistency in effect measures or validation frameworks to support such evaluation. This approach reflects the exploratory nature of the current literature on urinary biomarkers in Parkinson’s disease, where variability in study design and reporting remains a significant challenge. Therefore, the emphasis of this review was placed on identifying convergent biological patterns and integrating findings within a pathophysiological framework, rather than on quantitative comparison or hierarchical grading of evidence.

### 2.7. Data Synthesis

A qualitative and thematic synthesis of the data was conducted. Identified urinary biomarkers were grouped according to their genetic, proteomic, metabolomic, or pathophysiological relevance, including markers related to mitochondrial dysfunction, oxidative stress, lysosomal pathways, neuroinflammation, hormonal regulation, and gut–brain axis interactions. Given the heterogeneity of the data, meta-analysis was not feasible.

## 3. Results and Discussion

**Figure 2:**
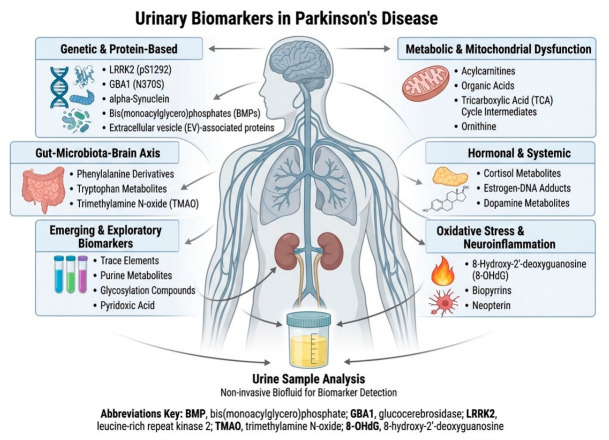
Conceptual framework illustrating the systemic organization of urinary biomarkers in PD. Urinary biomarkers were grouped into six interconnected pathophysiological domains reflecting molecular alterations associated with PD, including genetic and protein-based changes, metabolic and mitochondrial dysfunction, oxidative stress and neuroinflammation, gut–brain-axis-related metabolites, hormonal and systemic alterations, and emerging exploratory biomarkers. The central node highlights PD as a systemic disorder, with urine serving as an integrative molecular readout of these processes. BMP, endo-lysosomal phospholipid, bis(monoacylglycerol)phosphate; EVs, extracellular vesicles; GBA1, Glucocerebrosidase; 8-OHDG, 8-hydroxydeoxyquanosine; LRRK2, Leucine-rich repeat kinase 2 or dardarin; TCA, Tricarboxylic acid cycle; TMAO, Trimethylamine N-oxide.

The present review was organized into six thematic domains reflecting major pathophysiological processes implicated in PD to facilitate interpretation of the heterogeneous literature on urinary biomarkers. These domains encompass genetic and protein-based alterations, metabolic and mitochondrial dysfunction, oxidative stress and neuroinflammation, gut–brain-axis-related metabolites, hormonal and systemic changes, as well as emerging exploratory biomarkers. This framework allows a structured integration of evidence while highlighting the systemic and interconnected nature of urinary molecular signatures in PD. A schematic overview of this organization is provided in Figure 2.

### 3.1. Genetic and Protein-Based Urinary Biomarkers

An overview of the main genetic and protein-based urinary biomarkers investigated in PD, along with their methodological approaches and clinical relevance, is summarized in Table 1.

#### 3.1.1. Leucine-Rich Repeat Kinase 2 (LRRK2)

**Figure 3:**
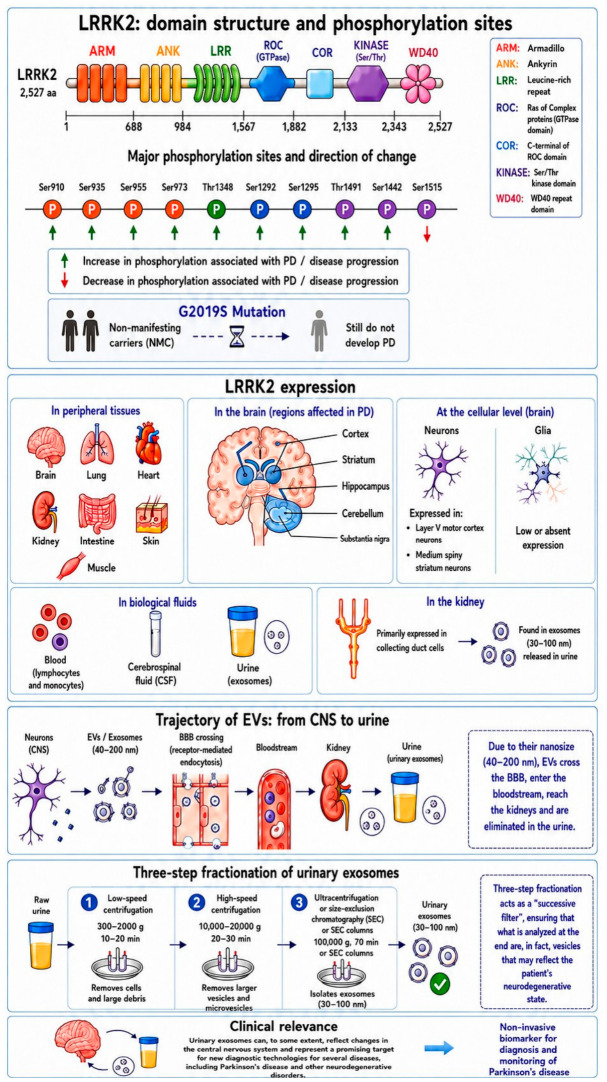
Leucine-Rich Repeat Kinase 2 (LRRK2) is the gene that encodes a 2527 amino acid protein containing two functional enzymatic domains, the GTPase and Ser/Thr kinase domains, and several protein–protein interaction domains, such as the armadillo, ankyrin, leucine-rich repeat (LRR), and WD4014 domains. Increased levels of LRRK2 protein occur in the prefrontal cortex of PD patients as the disease progresses, raising questions about whether LRRK2 detection could be used as a prognostic biomarker for the disease. However, interestingly, some individuals with the G2019S mutation, known as the non-manifesting carrier (NMC) group, do not yet develop PD. LRRK2 expression is described in various tissues, including brain, lung, heart, kidney, intestine, skin, and muscle. In the brain, it is expressed in areas clinically affected by Parkinson’s disease, such as the cortex, striatum, hippocampus, cerebellum, and substantia nigra. At the cellular level, it is expressed in neurons of the motor cortex of layer V and neurons of the middle spiny striatum; in glial cells, its expression is low or absent. In biological fluids, it is expressed in blood (in lymphocytes and monocytes), cerebrospinal fluid, and urine. In the kidney, LRRK2 is mainly expressed in collecting duct cells and is found in exosome isolates, i.e., cell-derived vesicles with a diameter of 30–100 nm, which can be released in the urine. Three-step fractionation acts as a “successive filter,” ensuring that what is being analyzed at the end are, in fact, the vesicles that may reflect the patient’s neurodegenerative state. Thanks to their nanometric size (40–200 nm), EVs can cross the endothelial cells of the blood-brain barrier (BBB) via receptor-mediated endocytosis, enter the bloodstream, reach the kidneys, and be eliminated in the urine. Therefore, urinary exosomes can, to some extent, reflect changes in the central nervous system, making them a target for new diagnostic technologies for various diseases, including Parkinson’s disease.

Genome-wide association studies (GWAS) have identified approximately 20 genes associated with hereditary PD, which accounts for about 10% of disease prevalence [29]. Among these, LRRK2 has emerged as one of the most extensively investigated genes due to its strong association with both familial and sporadic forms of PD [30]. LRRK2 encodes a large multidomain protein containing GTPase and serine/threonine kinase domains, as well as several protein–protein interaction domains, including armadillo, ankyrin, leucine-rich repeat, and WD40 domains (Figure 3).

The most common pathogenic mutation, G2019S, located in the kinase domain, is particularly prevalent in Caucasian populations and is associated with an approximately two- to three-fold increase in kinase activity [31], resulting in toxic gain-of-function effects [30]. Interestingly, the penetrance of this mutation is incomplete and age-dependent, ranging from about 28% at age 59 to 74% at age 79 [32,33], which highlights the importance of molecular biomarkers capable of distinguishing disease onset and progression (Figure 3).

LRRK2 is expressed in multiple tissues, including the brain, kidney, lung, heart, intestine, skin, and muscle. Within the kidney, LRRK2 is predominantly expressed in collecting duct cells and is released into urine through extracellular vesicles (EVs), particularly exosomes with diameters ranging from 30 to 100 nm. As such, urinary EVs have become a relevant source for investigating LRRK2-related biomarkers in PD. Initial studies evaluating total LRRK2 levels in urinary exosomes reported considerable interindividual variability, limiting their diagnostic utility when measured alone [20]. However, subsequent investigations demonstrated that phosphorylated forms of LRRK2, particularly pS1292-LRRK2, exhibit greater consistency and disease relevance. Elevated levels of pS1292-LRRK2 in urinary exosomes were observed in individuals carrying the G2019S mutation with PD compared with non-manifesting carriers and healthy controls [21,27]. Moreover, increased pS1292-LRRK2 levels were also reported in idiopathic PD and correlated with cognitive impairment severity [21] (Figure 3).

Beyond LRRK2 itself, LRRK2 substrates, including Rab GTPases, have also been proposed as urinary biomarkers. Reduced phosphorylation of Rab10 and altered levels of Rab8, pS910-LRRK2, and pS935-LRRK2 in urinary EVs have been associated with PD, with Rab phosphorylation appearing more sensitive to changes in LRRK2 kinase activity than total LRRK2 expression [26] (Figure 3).

#### 3.1.2. Glycocerebrosidase (GBA) and Lysosomal Pathways

Mutations in the GBA gene, which encodes the lysosomal enzyme glucocerebrosidase (GCase), represent one of the most common genetic risk factors for PD [15]. While homozygous mutations cause Gaucher disease, heterozygous GBA variants significantly increase PD susceptibility and are associated with earlier disease onset and cognitive decline [15].

Proteomic analyses of urine have revealed distinct molecular signatures associated with GBA-related PD. It was demonstrated that urinary proteomic profiles differ substantially between individuals carrying pathogenic mutations in LRRK2 and GBA, suggesting that these genetic variants affect largely independent biological pathways [15]. In carriers of GBA mutations, significant elevations in urinary levels of proteins such as intercellular adhesion molecule 1 (ICAM1), adenosyllocysteinase (AHCY), and stomatin (STOM) were observed. In contrast, lysosomal enzymes and proteins related to glycosphingolipid metabolism exhibited divergent expression patterns compared with carriers of LRRK2 mutations. Interestingly, the reduction in levels of proteins associated with glycosphingolipid metabolism, including GM2 activator (GM2A), was more pronounced in carriers of pathogenic GBA, corroborating the role of lysosomal dysfunction in PD pathogenesis beyond classical α-synuclein aggregation [24].

#### 3.1.3. Bis(monoacylglycerol)phosphate (BMP)

The BMP is a negatively charged phospholipid enriched in late endosomes and lysosomes, where it facilitates the activity of luminal hydrolases. Multiple studies have demonstrated that urinary BMP and its isoforms are elevated in individuals carrying LRRK2 mutations, particularly G2019S and R1441G/C, as well as VPS35 D620N mutations [22]. Further reported that specific BMP isoforms, including 2,2′-di-18:1-BMP and di-22:6-BMP, were significantly increased in LRRK2 mutation carriers and were modestly but significantly higher in PD patients compared with non-manifesting carriers [18]. Importantly, higher urinary levels of di-22:6-BMP were associated with worse cognitive performance, as assessed by the Montreal Cognitive Assessment. Genetic association analyses have reinforced these findings. It was demonstrated that single-nucleotide polymorphisms corresponding to LRRK2 G2019S and GBA1 N370S variants were among the strongest genetic determinants influencing urinary BMP levels, supporting BMP as a downstream readout of lysosomal and LRRK2-related dysfunction [19].

#### 3.1.4. Alpha-Synuclein

Alpha-synuclein, encoded by the SNCA gene, is a central protein in PD pathology and exists in multiple conformational states, including monomeric, oligomeric, and fibrillar forms. While total α-synuclein levels in urine do not consistently differ between PD patients and controls, conformationally specific species appear to hold greater diagnostic relevance. Increased levels of fibrillar α-synuclein in urine samples from PD patients were reported, whereas oligomeric α-synuclein levels were reduced [25]. Although these changes were not correlated with disease stage, the findings highlighted the importance of α-synuclein conformational state in biomarker development. More recently, employed surface-based fluorescence intensity distribution analysis (sFIDA) and demonstrated significantly elevated levels of α-synuclein aggregates in the urine of patients with PD and individuals with isolated REM sleep behavior disorder (iRBD), a recognized prodromal condition of PD [24]. These findings suggest that pathological α-synuclein species may be detectable in urine even before the onset of classical motor symptoms.

#### 3.1.5. Extracellular Vesicle-Associated Proteins

Urinary extracellular vesicles provide a stable and biologically enriched source of CNS-related proteins. The three-stage differential centrifugation protocol ensures rigorous isolation of extracellular vesicles (EVs) and elimination of urinary contaminants. The initial low-speed stage removes macroscopic cells and debris; the second stage eliminates protein aggregates and uromodulin polymers that can interfere with sample purity; and the final ultracentrifugation isolates the EV population of interest. This sequential approach is critical in biomarker research for Parkinson’s disease, as it enables the enrichment of vesicles of neural origin while minimizing analytical interference caused by non-vesicular proteins abundant in urinary fluid. Several studies have identified EV-associated proteins as potential urinary biomarkers for PD.

EVs exhibit different charges, display dynamic changes in number and content in response to physiological and environmental conditions. They can function as transporters of biomolecules, including lipids, proteins, second messengers, mRNA, miRNA, and fragments of cellular organelles, protecting them from external factors such as nucleases, proteases, and other degradative enzymes. The cargo incorporated into EVs can be transferred to target cells through ligand–receptor interaction, fusion, and/or internalization [34]. Owing to their nanoscale size and lipid bilayer structure, extracellular vesicles have been shown to traverse biological barriers, including the blood–brain barrier, and can ultimately be filtered by the kidneys and excreted in urine, thereby serving as peripheral carriers of CNS-related molecular information. Reported elevated levels of synaptosome-associated protein 23 (SNAP23) and calbindin (CALB1) in urinary EVs from PD patients [28]. Further identified multiple EV-associated proteins capable of distinguishing idiopathic PD from isolated Rapid Eye Movement (REM) sleep behavior disorder and predicting disease severity and future phenoconversion [23].

While DP-GBA is strongly associated with enzymatic dysfunction and substrate accumulation, DP-LRRK2 is correlated with alterations in membrane trafficking and extracellular vesicle (EV) secretion. LRRK2 is highly expressed in the renal epithelium and regulates endocytosis and autophagy processes. In carriers of pathogenic mutations, a urinary proteomic signature enriched with vesicular transport proteins and EV components has been observed, suggesting that urine may capture the impact of LRRK2 kinase hyperactivity on the endolysosomal system [28].

#### 3.1.6. Integrative Perspective on Genetic and Protein-Based Urinary Biomarkers

Taken together, the genetic and protein-based urinary biomarkers discussed in this section reveal a convergent pathophysiological framework centered on lysosomal dysfunction, altered kinase signaling, and protein aggregation, which are key processes in PD. Variants in LRRK2 and GBA influence downstream molecular signatures detectable in urine, including phosphorylation patterns of LRRK2, alterations in BMP isoforms, and changes in EV-associated protein cargo.

Importantly, the detection of pathological α-synuclein species and disease-related proteins in urinary extracellular vesicles supports the concept that urine can reflect central nervous system-related molecular alterations, despite its peripheral origin. Collectively, these findings suggest that panels combining genetic context with protein-based urinary biomarkers may provide greater diagnostic and prognostic value than single markers alone, reinforcing the potential of urine as a non-invasive matrix for PD biomarker discovery.

### 3.2. Metabolic Pathways and Mitochondrial Dysfunction

The main urinary metabolites related to mitochondrial dysfunction and altered energy metabolism in PD, as identified across metabolomic studies, are summarized in Table 2.

#### 3.2.1. Mitochondrial Dysfunction and Energy Metabolism

Mitochondrial dysfunction is a central feature in the pathophysiology of PD and is closely linked to dopaminergic neurodegeneration. In PD, α-synuclein oligomers accumulate at mitochondrial membranes, where they interfere with ATP synthase activity, disrupt calcium homeostasis, impair mitophagy, and alter mitochondrial dynamics, ultimately leading to increased production of reactive oxygen species (ROS) and bioenergetic failure [40].

Although PD is not classified as a primary disorder of fatty acid β-oxidation, increasing evidence indicates that mitochondrial dysfunction in PD affects lipid metabolism and energy production. Acylcarnitines, which facilitate the transport of fatty acids into mitochondria, have emerged as indirect indicators of mitochondrial impairment. Elevated urinary levels of hydroxylaurylcarnitine, hydroxyhexanoicarnitine, and malonylcarnitine were reported in patients with idiopathic PD, suggesting failure of the mitochondrial carnitine transport system and compromised β-oxidationReported [36].

#### 3.2.2. Organic Acids and Krebs Cycle Intermediates

**Figure 4:**
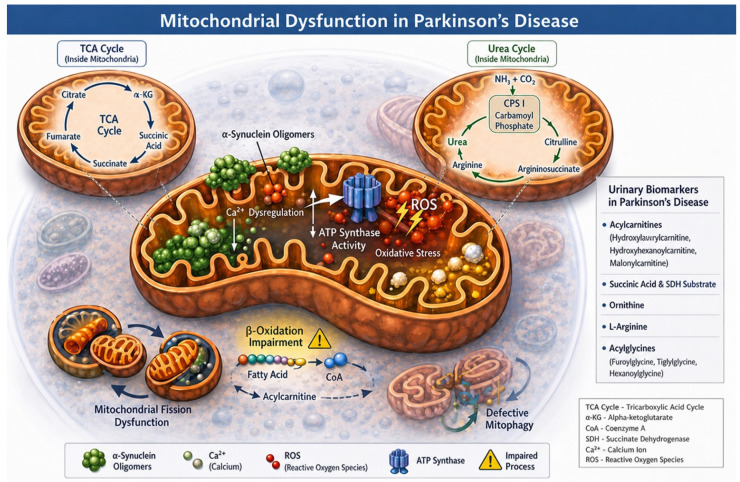
Mitochondrial dysfunction is a central feature in the pathophysiology of Parkinson’s disease and is closely linked to dopaminergic neurodegeneration, as α-synuclein oligomers accumulate in mitochondrial membranes, where they interfere with ATP synthase activity, impair calcium homeostasis, promote mitochondrial fission dysfunction and defective mitophagy, completely altering mitochondrial dynamics, ultimately leading to increased production of reactive oxygen species (ROS) and bioenergetic failure. This image shows the main biomolecules found in urine samples from the studies described in this review that demonstrate mitochondrial dysfunction. TCA Cycle—Tricarboxylic Acid Cycle; α-KG—Alpha-Ketoglutarate; CoA—Coenzyme A; SDH—Succinate Dehydrogenase Enzyme; Ca^2+^—Ionic Calcium; ROS—Reactive Oxygen Species.

Metabolomic analyses have identified alterations in several organic acids and Krebs cycle intermediates in the urine of PD patients. Succinic acid, a key intermediate of the tricarboxylic acid (TCA) cycle and a substrate of succinate dehydrogenase (Complex II of the mitochondrial respiratory chain), has been consistently reported as altered in PD (Figure 4). Reported [37,38] increased urinary levels of succinic acid in patients with intermediate and advanced stages of PD. Although Complex I dysfunction is more commonly implicated in PD, impairment of Complex II may also contribute to mitochondrial inefficiency, reduced ATP production, and enhanced oxidative stress [41]. Beyond succinate, several additional organic acids involved in intermediary metabolism have been reported as altered in the urine of PD patients, reflecting broader disturbances in mitochondrial and lysosomal metabolic pathways [39]. These metabolites are typically associated with inborn errors of metabolism, raising the possibility that shared lysosomal and mitochondrial pathways may underlie both metabolic disorders and certain forms of Parkinsonism [42].

The metabolomic study [35] identified elevated levels of ornithine in the urine of patients with PD. Ornithine, an amino acid widely known for its importance in the urea cycle, is also a precursor in the synthesis of polyamines, which include spermidine, spermine, and putrescine [43,44]. Polyamines are poly-cationic organic molecules that are essential for various cellular processes, such as cell growth and proliferation, DNA and RNA stability, regulation of autophagy, modulation of ion channels and receptors, and also act as antioxidants [43,44].

Polyamines, especially spermidine, have been extensively studied for their ability to activate autophagy and promote the removal of protein aggregates and dysfunctional organelles. Spermidine activates autophagy through complex mechanisms, including inhibition of the mTORC protein and acetylation of autophagic proteins. By inducing autophagy, spermidine helps the cell remove aggregated proteins such as altered alpha synuclein and damaged mitochondria in PD [43,44]. Alterations in ornithine metabolism may indicate dysregulation in the polyamine pathway and deficient autophagy, a condition present in PD [43,44].

L-arginine, a semi-essential amino acid that is converted to ornithine in the urea cycle (Figure 4) and polyamine biosynthesis, is also a substrate for another relevant pathway in PD, the nitric oxide synthase (NOS) metabolic pathway [45]. In this pathway, arginine is converted into nitric oxide (NO) and citrulline by the action of nitric oxide synthase (NOS) enzymes. Under physiological conditions, the NO produced by nNOS and eNOS is essential for synaptic plasticity, regulation of cerebral blood flow, and neurotransmission [45]. However, in PD, the excess NO produced by iNOS can react with superoxide to form peroxynitrite, a highly toxic free radical that damages proteins, lipids, and DNA, generating nitrosative stress and neuroinflammation that directly contributes to the death of dopaminergic neurons in the substantia nigra [45]. Thus, altered ornithine levels may also reflect an imbalance in arginine bioavailability between metabolic pathways, due to the state of chronic neuroinflammation present in PD [45].

#### 3.2.3. Acylglycines and Fatty Acid Detoxification Pathways

Acylglycines are formed through the conjugation of fatty acids or organic acids with glycine, serving as an alternative detoxification route when mitochondrial β-oxidation is impaired. Elevated urinary levels of acylglycines, including furoylglycine, tiglylglycine, and hexanoylglycine, were reported by [37] in PD patients. Increased excretion of acylglycines may reflect systemic mitochondrial dysfunction, diversion of accumulating acyl-CoA intermediates, and compensatory detoxification mechanisms. Such metabolic alterations are consistent with impaired energy metabolism, increased oxidative stress, and neuroinflammation, all of which are hallmarks of PD progression [46]. Notably, elevated urinary levels of glutaric acid were also associated with intermediate and advanced stages of PD [37]. While glutaric acid accumulation is classically linked to glutaric aciduria type I, this finding raises intriguing questions about overlapping metabolic vulnerabilities affecting basal ganglia function and mitochondrial integrity in PD [47].

#### 3.2.4. Amino Acid-Related Metabolic Alterations

Beyond lipid and organic acid metabolism, PD-related mitochondrial dysfunction also affects amino acid metabolism. Metabolomic studies have reported altered urinary levels of glycine derivatives, histidine-related compounds, and branched-chain amino acid metabolites in PD patients [35,36,37]. These alterations may reflect disrupted nitrogen balance, impaired detoxification pathways, and altered neurotransmitter precursor availability. Given the close metabolic coupling between amino acid catabolism and mitochondrial energy production, such changes further support the presence of widespread metabolic dysregulation in PD.

#### 3.2.5. Integrative Perspective on Metabolic Dysregulation and Mitochondrial Dysfunction

The urinary metabolic alterations described in this section converge on a common framework of mitochondrial dysfunction, impaired energy metabolism, and compensatory detoxification pathways in PD. Changes in acylcarnitines, organic acids, acylglycines, and Krebs cycle intermediates suggest systemic bioenergetic stress that extends beyond the central nervous system and is detectable in peripheral biofluids (Figure 4). Although these metabolic signatures lack disease specificity when considered individually, their combined assessment may provide valuable insights into disease progression and mitochondrial vulnerability. Integrating metabolic biomarkers with genetic and protein-based urinary markers may therefore enhance the sensitivity and interpretability of non-invasive biomarker panels for PD.

### 3.3. Oxidative Stress and Neuroinflammation

Key urinary biomarkers associated with oxidative stress and neuroinflammation in PD, including markers of DNA damage and immune activation, are summarized in Table 3.

#### 3.3.1. Oxidative DNA Damage

Oxidative stress is a central mechanism in PD pathophysiology and is closely linked to mitochondrial dysfunction and dopaminergic neurodegeneration. Reactive oxygen species (ROS), such as hydroxyl radicals and hydrogen peroxide, can interact with DNA bases, particularly guanine, leading to the formation of 8-hydroxy-2′-deoxyguanosine (8-OHdG), a widely used marker of oxidative DNA damage that is continuously repaired and excreted in urine. It was demonstrated that urinary 8-OHdG levels were significantly elevated in patients with PD compared with healthy controls and increased progressively with disease stage [48]. However, similar elevations were observed in patients with multiple system atrophy (MSA), indicating limited disease specificity. In contrast, it was reported higher urinary 8-OHdG levels in early-stage PD compared with more advanced stages and identified a negative correlation with cumulative levodopa intake, highlighting variability in the relationship between oxidative damage and disease progression [49]. Collectively, these observations indicate that urinary markers of oxidative damage, such as 8-OHdG, primarily reflect systemic redox imbalance in PD rather than disease-specific pathology, highlighting the importance of integrative biomarker interpretation.

#### 3.3.2. Bilirubin Metabolism and Biopyrrins

Bilirubin acts as an endogenous antioxidant, but its interaction with ROS generates oxidative metabolites known as biopyrrins, which are excreted in urine. Elevated urinary biopyrrin levels have been proposed as indicators of increased oxidative stress in vivo. [50] reported significantly increased urinary biopyrrin levels in patients with idiopathic PD across all disease stages, using two independent analytical methodologies. These findings support urinary biopyrrins as sensitive markers of systemic oxidative stress, although their specificity for PD remains uncertain.

#### 3.3.3. Immune Activation and Inflammatory Markers

Neuroinflammation is a key contributor to PD progression, driven largely by microglial activation and the release of pro-inflammatory mediators. Neopterin, a stable pteridine derivative produced by activated macrophages in response to interferon-γ, serves as a marker of cellular immune activation and can be measured in urine. Evaluated urinary neopterin levels in patients with idiopathic REM sleep behavior disorder and PD and found increased neopterin concentrations associated with worsening olfactory dysfunction, a non-motor feature commonly linked to early neurodegenerative processes [51]. These findings suggest that urinary neopterin may reflect immune activation and inflammatory processes associated with PD-related neurodegeneration.

#### 3.3.4. Neuroinflammatory Modulators and Metabolites

In addition to classical inflammatory markers, certain metabolites with known anti-inflammatory or pro-inflammatory properties have been implicated in PD-related neuroinflammation. Vanillic acid, a phenolic compound with antioxidant and anti-inflammatory effects, has been shown in experimental models to suppress lipopolysaccharide-induced activation of glial cells through inhibition of NF-κB and MAPK signaling pathways [52]. In a human metabolomic study, [39] reported elevated urinary levels of vanillic acid in PD patients compared with healthy controls. Although the mechanistic relevance of vanillic acid in human PD remains incompletely understood, these findings raise the possibility that urinary metabolites may reflect compensatory or modulatory responses to chronic neuroinflammation.

#### 3.3.5. Integrative Perspective on Oxidative Stress and Neuroinflammation

Urinary biomarkers related to oxidative stress and immune activation highlight the systemic nature of redox imbalance and neuroinflammatory processes in PD. Markers such as 8-OHdG, biopyrrins, neopterin, and inflammation-associated metabolites provide indirect but accessible indicators of molecular damage and immune responses associated with disease progression. While these biomarkers individually lack specificity for PD, their combined evaluation may enhance sensitivity for detecting disease-related pathological processes, particularly when integrated with genetic, proteomic, and metabolic urinary signatures. Such multidimensional approaches may improve the utility of non-invasive biomarker panels for monitoring PD progression and therapeutic responses.

### 3.4. Gut–Brain Axis and Microbiota-Derived Metabolites

Urinary metabolites related to gut microbiota activity and gut–brain axis interactions reported in PD are summarized in Table 4.

#### 3.4.1. Microbiota–Gut–Brain Axis

Growing evidence supports the involvement of the microbiota–gut–brain axis in PD pathogenesis. Gastrointestinal symptoms, such as constipation, often precede motor manifestations, suggesting that intestinal dysfunction may represent an early event in disease development [55,56,57,58,59]. Parkinson’s disease-associated microbiota shows reduced butyrate production and increased harmful proteolytic metabolites, changes linked to constipation and potentially involved in disease pathophysiology [55,56,57,58,59]. Bidirectional communication between the gut microbiota and the central nervous system occurs through neural, immune, and metabolic pathways, including signaling via the vagus nerve [54,55]. Altered microbial composition and metabolic output may influence brain physiology through the production of neuroactive and immunomodulatory metabolites that reach the systemic circulation and are ultimately excreted in urine, making this biofluid a valuable source for investigating gut-derived biomarkers in PD.

#### 3.4.2. Phenylalanine and Phenylacetylglutamine Pathway

Several urinary metabolites related to phenylalanine metabolism have been associated with PD. Reported [36] increased urinary levels of phenylacetic acid, phenylacetylglutamine, and trimethylamine N-oxide (TMAO) in patients with idiopathic PD compared with healthy controls. In a subsequent study, the same group observed that phenylalanine and its derivatives were particularly prevalent in the early stages of the disease [37]. Phenylacetylglutamine is produced both through host metabolism and by specific gut bacterial species, linking its urinary excretion to microbial activity. Alterations in intestinal permeability and microbiota composition may enhance systemic exposure to these metabolites, contributing to inflammation and oxidative stress relevant to PD pathophysiology [57]. Consistent with these findings, altered urinary phenylalanine levels have also been reported by [35].

#### 3.4.3. Tryptophan Metabolism and Kynurenine Pathway

Tryptophan metabolism represents a major interface between the gut microbiota and host neurobiology. Three primary metabolic routes are recognized: the indole pathway mediated by intestinal microbiota, the kynurenine pathway, and the serotonin pathway. Indole metabolites, produced exclusively by gut bacteria, can act as ligands for the aryl hydrocarbon receptor (AhR), influencing immune responses and CNS function. They [54] developed an LC–MS/MS method to quantify 21 tryptophan metabolites in urine and reported that indole-3-acetic acid was the only metabolite significantly altered in PD patients compared with controls. In contrast, [53] identified urinary kynurenine as a potential biomarker in early-stage PD, although this study relied on an ELISA-based approach that did not discriminate among downstream kynurenine metabolites. Metabolomic analyses by [36,50] further demonstrated alterations in the kynurenine/kynurenic acid ratio, as well as stage-dependent changes in quinurenine, hydroxytryptophan, xanthuric acid, and tryptophan itself. These findings highlight both methodological variability and the complexity of interpreting tryptophan-derived urinary biomarkers in PD.

#### 3.4.4. Trimethylamine N-Oxide (TMAO) and Microbial Metabolites

The TMAO is produced through the microbial metabolism of dietary choline and L-carnitine, followed by hepatic oxidation. Elevated urinary TMAO levels have been reported in PD patients [36], suggesting altered microbial metabolism and host–microbe interactions. TMAO has been implicated in systemic inflammation and vascular dysfunction, and although its direct role in PD remains unclear, its detection in urine underscores the contribution of gut-derived metabolites to systemic biochemical alterations associated with neurodegeneration [57].

#### 3.4.5. Integrative Perspective on Gut–Brain-Axis-Related Urinary Biomarkers

**Figure 5:**
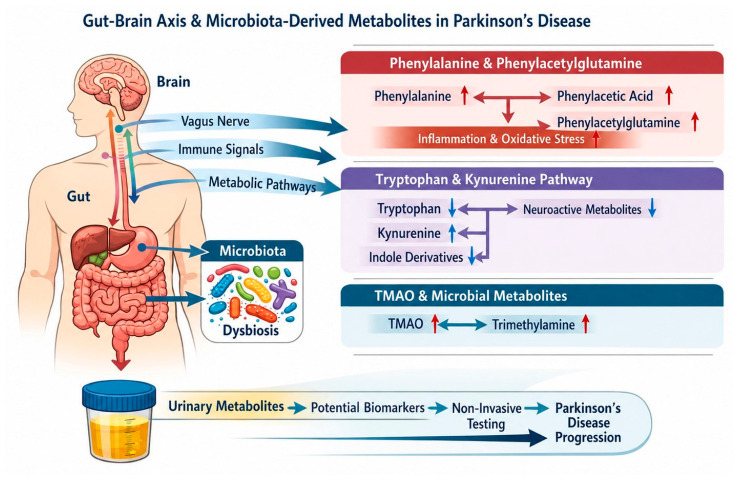
Growing evidence supports the involvement of the gut–brain axis in the pathogenesis of Parkinson’s disease. Gastrointestinal symptoms, such as constipation, often precede motor manifestations, suggesting that intestinal dysfunction may represent an early event in the development of the disease. Bidirectional communication between the gut microbiota and the CNS—central nervous system—occurs through neural, immunological, and metabolic pathways, including signaling via the vagus nerve. Altered microbial composition and metabolic output can influence brain physiology through the production of neuroactive and immunomodulatory metabolites that reach the systemic circulation and are ultimately excreted in the urine, making this biofluid a valuable source for investigating gut-derived biomarkers in PD. In this context, urinary metabolites related to phenylalanine metabolism have been associated with PD, such as phenylacetic acid, phenylacetylglutamine, trimethylamine oxide (TMAO), and tryptophan metabolites.

Urinary metabolites derived from phenylalanine and tryptophan metabolism, as well as microbial products such as phenylacetylglutamine and TMAO, support a functional link between gut microbiota dysbiosis and PD-related pathophysiology (Figure 5). These metabolites likely reflect a combination of altered microbial composition, intestinal permeability, and host metabolic responses. Although individual metabolites show variability across studies and limited disease specificity, their integration into broader metabolic and inflammatory biomarker panels may provide insights into early and prodromal stages of PD. Urine-based assessment of gut–brain-axis-related metabolites therefore represents a promising, non-invasive approach for exploring systemic contributions to neurodegeneration (Figure 5).

### 3.5. Hormonal and Systemic Biomarkers

An overview of hormonal and systemic urinary biomarkers associated with PD, including estrogen-derived metabolites, cortisol-related compounds, and dopamine-related metabolites, is presented in Table 5.

#### 3.5.1. Estrogen Metabolism and Catechol Estrogens

Sex-related differences in PD incidence and progression have long suggested a modulatory role for steroid hormones in disease susceptibility and progression, particularly estrogens. Dopaminergic neurodegeneration in PD is accompanied by complex interactions between dopamine metabolism and estrogen-derived compounds, especially catechol estrogens. Catechol estrogens, which are metabolites of estradiol and estrone, share structural and metabolic similarities with dopamine. Mechanistically, both dopamine and catechol estrogens can undergo bioactivation to quinone intermediates capable of reacting with DNA and other macromolecules, leading to the formation of depurination DNA adducts.

Under acidic or oxidative conditions, dopamine quinones may react with nucleophilic sites in DNA, similarly to estrogen-derived catechol quinones, resulting in apurinic sites and genomic instability. In this context, ref. [60] demonstrated that urinary levels of estrogen-derived depurination DNA adducts, including 4-OHE1(E2)-1-N3Ade and 4-OHE1(E2)-1-N7Gua, differed significantly between PD patients and healthy controls. These findings support the hypothesis that dysregulated estrogen metabolism may contribute to dopaminergic vulnerability through mechanisms that parallel dopamine quinone-mediated oxidative and genotoxic stress, reinforcing the relevance of urinary estrogen metabolites as systemic indicators of PD-related molecular stress.

Beyond estrogens, other steroid hormones may also contribute to systemic vulnerability in PD. Dysregulation of the hypothalamic–pituitary–adrenal (HPA) axis has been implicated in PD, potentially linking chronic stress responses to neurodegeneration. Cortisol, the primary glucocorticoid hormone, influences neuronal energy metabolism, mitochondrial function, and inflammatory signaling. Metabolomic analyses have reported elevated urinary levels of cortisol and related steroid metabolites, including 11-deoxycortisol and 21-deoxycortisol, in PD patients [36]. Together, these findings suggest that altered steroid hormone metabolism—encompassing both estrogens and glucocorticoids—may represent a systemic marker of stress-related vulnerability and disease progression in PD, although confounding effects of medication and circadian variability must be considered.

From a biomarker perspective, the detection of estrogen-derived catechol metabolites and depurination DNA adducts in urine should not be interpreted solely as a reflection of sex hormone status, but rather as an indicator of systemic catechol-related redox and genotoxic stress. The biochemical parallels between dopamine quinone formation and estrogen-derived catechol quinones suggest convergent mechanisms of molecular vulnerability that extend beyond the central nervous system. In this context, urine represents an integrative matrix capable of capturing cumulative oxidative and genotoxic processes arising from dysregulated catechol metabolism, reinforcing the relevance of estrogen-related urinary biomarkers as part of a broader systemic signature of PD.

#### 3.5.2. Dopamine-Related Metabolites and Precursors

In PD, progressive loss of dopaminergic neurons leads to substantial alterations in dopamine synthesis and metabolism. Metabolomic profiling by [39] revealed elevated urinary levels of 3-methoxytyramine and N-acetyl-L-tyrosine, both of which are related to dopamine biosynthesis and catabolism. These metabolites may reflect compensatory changes in dopamine turnover or altered peripheral handling of dopamine precursors. Given that dopamine metabolism is tightly linked to oxidative stress and quinone formation, their urinary detection may indirectly mirror neurodegenerative processes occurring in the substantia nigra.

#### 3.5.3. Hypothalamic–Pituitary–Adrenal Axis and Cortisol

The hypothalamic–pituitary–adrenal (HPA) axis plays a central role in stress regulation and energy homeostasis, and its dysregulation has been implicated in PD. Chronic stress and altered glucocorticoid signaling may exacerbate neuronal vulnerability by impairing mitochondrial function and promoting neuroinflammation. Identified elevated urinary levels of cortisol, 11-deoxycortisol, and 21-deoxycortisol in PD patients, suggesting altered steroidogenesis and HPA axis activity [36]. These findings are consistent with reports indicating that long-term levodopa treatment and disease-related stress can influence cortisol dynamics [61,62]. Urinary cortisol metabolites may therefore serve as accessible indicators of systemic stress responses and neuroendocrine alterations associated with PD progression.

#### 3.5.4. Integrative Perspective on Hormonal and Systemic Urinary Biomarkers

**Figure 6:**
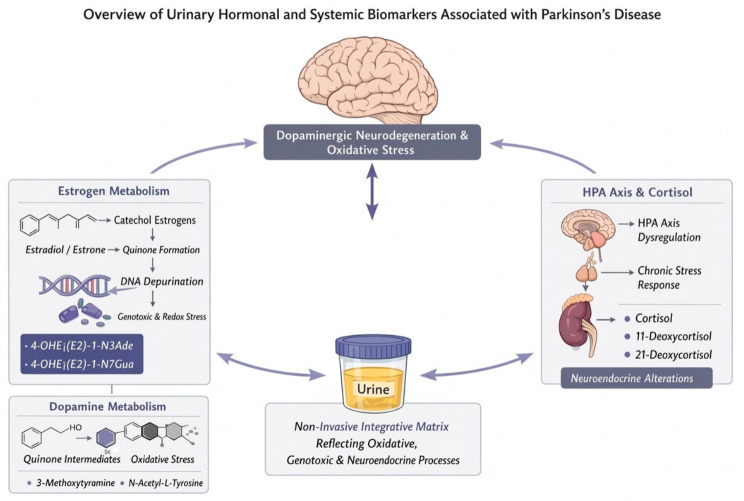
An overview of urinary hormonal and systemic biomarkers associated with Parkinson’s Disease, including estrogen-derived metabolites, cortisol-related compounds, and dopamine-related metabolites. Catecholestrogens exhibit structural and metabolic similarities to dopamine; both can undergo bioactivation into quinone intermediates capable of reacting with DNA and other macromolecules, leading to the formation of DNA adducts by depurination, including 4-OHE1(E2)-1-N3Ade and 4-OHE1(E2)-1-N7Gua. This suggests that dysregulated estrogen metabolism may contribute to dopaminergic vulnerability through mechanisms parallel to oxidative and genotoxic stress mediated by dopamine quinones. Dysregulation of the hypothalamic–pituitary–adrenal (HPA) axis has also been implicated in PD, potentially linking chronic stress responses to neurodegeneration, as reflected in elevated urinary levels of cortisol and related steroid metabolites, including 11-deoxycortisol and 21-deoxycortisol, in PD patients. Elevated urinary levels of 3-methoxytyramine and N-acetyl-L-tyrosine, both related to dopamine biosynthesis and catabolism, may reflect compensatory changes in dopamine turnover or alterations in the peripheral processing of dopamine precursors.

Alterations in estrogen metabolites, dopamine-related compounds, and cortisol derivatives highlight the contribution of endocrine and systemic regulatory pathways to PD pathophysiology. These hormonal biomarkers likely reflect a convergence of neurodegenerative processes, compensatory metabolic responses, and chronic stress exposure. Although hormonal markers alone lack specificity for PD, their integration with genetic, proteomic, and metabolic urinary biomarkers may improve the interpretation of disease-related systemic alterations (Figure 6). Such multidimensional approaches reinforce the potential of urine as a non-invasive matrix for capturing neuroendocrine and metabolic signatures relevant to PD (Figure 6).

### 3.6. Emerging and Exploratory Urinary Biomarkers

In addition to the more established classes of urinary biomarkers discussed above, a diverse set of emerging and exploratory molecular signatures has been reported in PD, reflecting systemic alterations associated with neurodegeneration. Altered metal homeostasis, including changes in urinary levels of copper, iron, zinc, and manganese, has been observed in PD patients and may contribute to oxidative stress, mitochondrial dysfunction, and protein aggregation. However, substantial interindividual variability and overlap with other neurological and systemic conditions limit the specificity of metal-based urinary biomarkers [35].

Dysregulation of purine metabolism has also been consistently reported in urinary metabolomic studies of PD, particularly involving uric acid, xanthine, and hypoxanthine [35,36]. While uric acid is recognized for its antioxidant properties and inverse association with PD risk, urinary purine metabolites are influenced by diet, renal function, and comorbidities, complicating their interpretation as disease-specific markers. Additional exploratory findings include alterations in glycosylation-related metabolites and carbohydrate metabolism, suggesting broader metabolic remodeling in PD [39]. Furthermore, changes in monoaminergic and noradrenergic metabolites have been reported and may reflect degeneration beyond the dopaminergic system, contributing to non-motor symptoms such as autonomic dysfunction and cognitive impairment [39].

In the study by Luan et al. (2015b), altered levels of pyridoxic acid in urine were associated with the intermediate and advanced stages of PD. Pyridoxic acid is the final metabolite of vitamin B6 (pyridoxine) that is excreted in the urine. The assessment of vitamin B6 in the body generally involves measuring both pyridoxal 5′-phosphate (the active form) and pyridoxic acid [63].

Levodopa is the most commonly used medication in the treatment of PD, and its metabolism is interconnected with that of vitamin B6. At very high doses of vitamin B6, or in some older formulations, vitamin B6 can increase the metabolism of levodopa before it reaches the brain and reduce its effectiveness. Therefore, DOPA-decarboxylase inhibitors (such as carbidopa or benserazide) are administered along with levodopa, as they prevent the breakdown of levodopa outside the CNS and this problem is minimized [63]. Patients with PD, especially those on prolonged levodopa treatment, may have deficiencies in B vitamins, including B6 and B12. These deficiencies can accelerate the development of levodopa-induced dyskinesias and on-off fluctuations, as well as cause peripheral neuropathies. Thus, measuring pyridoxic acid in urine could, in theory, help identify these deficiencies and assist in ensuring adequate vitamin B6 levels in the body [63].

Collectively, these exploratory urinary biomarkers capture complementary aspects of metabolic, oxidative, and neurotransmitter-related dysregulation in PD but remain insufficiently validated for standalone clinical application. Their integration into multimodal biomarker panels, alongside genetic, proteomic, metabolic, inflammatory, and hormonal markers, may enhance sensitivity for characterizing disease heterogeneity. Future studies should prioritize longitudinal designs, standardized analytical platforms, and integration with clinical phenotyping to clarify their translational potential.

Rather than representing isolated or peripheral findings, emerging urinary biomarkers may be viewed as molecular indicators of the biological heterogeneity that characterizes PD. Alterations in metal homeostasis, purine metabolism, carbohydrate processing, and monoaminergic metabolites likely reflect interconnected metabolic and oxidative stress responses that vary across individuals and disease stages. Although these markers lack sufficient specificity for standalone clinical use, their inclusion in integrative, multi-domain biomarker panels may enhance sensitivity for capturing disease-related systemic remodeling. In this sense, exploratory urinary biomarkers should be considered complementary components within a broader, systems-level approach to PD biomarker discovery.

## 4. Final Considerations

This review highlights the growing evidence supporting urine as a valuable and non-invasive biofluid for the investigation of molecular alterations associated with PD. Genetic, proteomic, metabolic, inflammatory, hormonal, and microbiota-derived urinary biomarkers collectively reflect systemic manifestations of key pathophysiological processes, including mitochondrial dysfunction, lysosomal impairment, oxidative stress, neuroinflammation, and altered neurotransmitter metabolism. Despite these advances, most urinary biomarkers identified to date lack sufficient specificity or validation when considered individually, underscoring the limitations of single-marker approaches in a biologically heterogeneous disorder such as PD. Variability in analytical platforms, study design, and disease stage further complicates direct comparisons across studies.

In this context, integrated omics-based strategies, particularly untargeted and targeted metabolomics and peptidomics/proteomics, emerge as promising tools for identifying combinatorial molecular signatures with improved sensitivity and specificity. High-resolution mass spectrometry-based approaches, including LC–MS/MS profiling of urinary metabolites, peptides, and extracellular vesicle cargo, may enable the discovery of robust biomarker panels capable of capturing disease complexity and progression.

## 5. Conclusions

Future studies should prioritize standardized analytical workflows, longitudinal cohort designs, and integration with clinical phenotyping to advance the translational potential of urinary biomarkers. Such approaches may ultimately support earlier diagnosis, improved disease stratification, and non-invasive monitoring of PD progression and therapeutic response.

## Figures and Tables

**Table 1:** Genetic and Protein-Based Urinary Biomarkers in PD.

Author (Year)	Study Design/Population	Biomarker Class	Urinary Biomarker(s)	Analytical Method	Main Findings	Clinical Relevance
Alcalay et al. (2020) [18]	Case–control (PD, NMC, HC)	Lipid/ Lysosomal	BMP isoforms (di-18:1, di-22:6)	LC–MS/MS	BMP ↑ in LRRK2-PD; di-22:6-BMP assoc. cognition	Disease severity
Fang et al. (2024) [19]	Genetic association study	Lipid/ Lysosomal	BMP	LC–MS/MS; GWAS	LRRK2 and GBA1 variants modulate BMP levels	Genetic modulation
Fraser et al. (2013) [20]	Case–control (PD, HC)	Genetic/ Protein (EV)	Total LRRK2	EV isolation; immunoblot	High interindividual variability; limited diagnostic value	Exploratory
Fraser et al. (2016b) [21]	Case–control (PD, NMC, HC)	Genetic/ Protein (EV)	pS1292-LRRK2	EV isolation; immunoblot	pS1292-LRRK2 ↑ (PD vs. NMC); assoc. cognitive impairment	Disease severity
Gomes et al. (2023) [22]	Case–control (PD, HC)	Lipid/ Lysosomal	BMP isoforms	LC–MS/MS	BMP ↑ in LRRK2 and VPS35 mutation carriers	Genetic stratification
Hallqvist et al. (2023) [23]	Cohort (iRBD, PD)	EV-associated proteins	EV protein panel	EV proteomics	Distinguishes PD from iRBD; predicts phenoconversion	Prodromal PD
Müller et al. (2025) [24]	Case–control (PD, iRBD, HC)	Protein	α-synuclein aggregates	sFIDA	α-syn aggregates ↑ (PD and iRBD)	Prodromal PD
Nam et al. (2020) [25]	Case–control (PD, HC)	Protein	Fibrillar and oligomeric α-synuclein	Immunoassay	Fibrillar α-syn ↑; oligomeric α-syn ↓ (PD)	Exploratory
Taymans et al. (2023) [26]	Case–control (PD, HC)	Genetic/ Protein (EV)	Rab10, Rab8, pS910-LRRK2, pS935-LRRK2	EV analysis; phospho-protein assays	Altered Rab phosphorylation; reflects LRRK2 kinase activity	Pathway activity
Virreira Winter et al. (2021) [15]	Cross-sectional (LRRK2-PD, GBA-PD, HC)	Proteomic	ICAM1, AHCY, STOM, GM2A	LC–MS/MS proteomics	Distinct urinary proteomic profiles by genetic background	Genetic stratification
Wang et al. (2017) [27]	Case–control (PD, HC)	Genetic/ Protein (EV)	pS1292-LRRK2	EV analysis; immunoassay	pS1292-LRRK2 ↑ (PD)	Diagnosis
Wang et al. (2019) [28]	Case–control (PD, HC)	EV-associated proteins	SNAP23, CALB1	EV proteomics	SNAP23 ↑; CALB1 ↑ (PD)	Diagnosis

PD, Parkinson’s disease; HC, healthy controls; NMC, non-manifesting carriers; iRBD, isolated rapid eye movement sleep behavior disorder; EV, extracellular vesicles; LRRK2, leucine-rich repeat kinase 2; GBA, glucocerebrosidase gene; BMP, bis(monoacylglycerol)phosphate; α-syn, alpha-synuclein; LC–MS/MS, liquid chromatography–tandem mass spectrometry; sFIDA, surface-based fluorescence intensity distribution analysis; Rab, Rat brain; ICAM1, Intercellular Adhesion Molecule 1; AHCY, S-Adenosylhomocysteine Hydrolase; STOM, Stomatin; GM2A, GM2 Ganglioside Activator; SNAP23, Synaptosome Associated Protein 23; CALB1, Calbindin 1; VPS35, Vacuolar Protein Sorting-associated protein 35. Arrows indicate the direction of change in PD compared with controls (↑ increased; ↓ decreased).

**Table 2:** Metabolic pathways and mitochondrial dysfunction-related urinary biomarkers in PD.

Author (Year)	Study Design/Population	Biomarker Class	Urinary Biomarker(s)	Analytical Method	Main Findings	Clinical Relevance
Kumari et al. (2020) [35]	Case–control (PD, HC)	Metabolomic	Amino acid derivatives	LC–MS/MS metabolomics	Amino acid metabolism altered	Metabolic remodeling
Luan et al. (2015a) [36]	Case–control (PD, HC)	Metabolomic; Mitochondrial	Acylcarnitines (C6-OH, C12-OH), hydroxyacids	GC–MS metabolomics	Acylcarnitines ↑; impaired β-oxidation	Metabolic dysregulation
Luan et al. (2015b) [37]	Case–control (PD, HC)	Metabolomic; Mitochondrial	Succinic acid, acylglycines, glutaric acid	GC–MS metabolomics	Succinic acid ↑; acylglycines ↑ (mid–advanced PD)	Disease progression
Michell et al. (2008) [38]	Case–control (PD, HC)	Metabolomic	Succinic acid	GC–MS metabolomics	Succinic acid ↑ (advanced PD)	Disease stage
Wang et al. (2023) [39]	Case–control (PD, HC)	Metabolomic	Organic acids (3,3-dimethylglutaric, orotic acid)	LC–MS/MS metabolomics	Organic acids altered (PD)	Exploratory

PD, Parkinson’s disease; HC, healthy controls; GC–MS, gas chromatography–mass spectrometry; LC–MS/MS, liquid chromatography–tandem mass spectrometry; TCA cycle, Tricarboxylic Acid Cycle. Arrows indicate the direction of change in PD compared with controls (↑ increased).

**Table 3:** Oxidative stress and neuroinflammation-related urinary biomarkers in PD.

Author (Year)	Study Design/Population	Biomarker Class	Urinary Biomarker(s)	Analytical Method	Main Findings	Clinical Relevance
Sato et al. (2005) [48]	Case–control (PD, HC)	Metabolomic	8-OHdG	HPLC-ECD/ELISA	8-OHdG ↑ in PD are positively correlated with disease duration and severity.	Oxidative stress
Seet et al. (2010) [49]	Case–control (PD, HC)	Metabolomic	8-OHdG and isoprostanes	LC-MS/MS	8-OHdG ↑ and 8-iso-PGF2α ↑ in the early phase of PD; negative correlation between 8-OHdG and the cumulative dose of levodopa.	Oxidative stress
Luan et al. (2015c) [50]	Case–control (PD, HC)	Metabolomic	Biopyrrins	LC–MS/MS	Significant increase in urinary biopyrrins in all stages of PD (early and advanced), reflecting the oxidative metabolism of bilirubin.	Oxidative stress
Campolo et al. (2016) [51]	Case–control (PD, HC)	Metabolomic	Neopterin	LC-MS/MS	PD with neopterin ↑, inversely associated with olfactory identification scores.	Neuroinflammation
Wang et al. (2023) [39]	Case–control (PD, HC)	Metabolomic	Vanillic acid	LC–MS/MS metabolomics	Vanillic acid ↑ in DP with HC.	Neuroinflammation

PD, Parkinson’s disease; HC, healthy controls; 8-OHdG, 8-hydroxy-2′-deoxyguanosine; 8-iso-PGF2α, 8-epi-prostaglandina F2α; GC–MS, gas chromatography–mass spectrometry; LC–MS/MS, liquid chromatography–tandem mass spectrometry. Arrows indicate the direction of change in PD compared with controls (↑ increased).

**Table 4:** Gut–brain axis and microbiota-derived urinary biomarkers in PD.

Author (Year)	Study Design/Population	Biomarker Class	Urinary Biomarker(s)	Analytical Method	Main Findings	Clinical Relevance
Bai et al. (2021) [53]	Case–control (early PD, HC)	Gut–brain axis	Kynurenine	ELISA	Kynurenine ↑ (early PD)	Early diagnosis
Chung et al. (2023) [54]	Case–control (PD, HC)	Gut–brain axis/Metabolomic	Indole-3-acetic acid	LC–MS/MS	Indole-3-acetic acid ↓ (PD)	Microbiota-related changes
Kumari et al. (2020) [35]	Case–control (PD, HC)	Metabolomic; Gut–brain axis	Phenylalanine, tryptophan metabolites	LC–MS/MS metabolomics	Aromatic amino acid metabolism altered	Exploratory
Luan et al. (2015a) [36]	Case–control (PD, HC)	Metabolomic; Gut–brain axis	Phenylacetic acid, phenylacetylglutamine, TMAO	GC–MS metabolomics	Phenylalanine-derived metabolites ↑ (PD)	Early metabolic changes
Luan et al. (2015b) [37]	Case–control (PD, HC)	Metabolomic; Gut–brain axis	Phenylalanine, quinurenine derivatives	GC–MS metabolomics	Phenylalanine-related metabolites altered (early PD)	Disease stage
Wang et al. (2023) [39]	Case–control (PD, HC)	Gut–brain axis/Metabolomic	Microbiota-derived metabolites	LC–MS/MS metabolomics	Gut-derived metabolites altered	Exploratory

PD, Parkinson’s disease; HC, healthy controls; LC–MS/MS, liquid chromatography–tandem mass spectrometry. Arrows indicate the direction of change in PD compared with controls (↑ increased; ↓ decreased).

**Table 5:** Hormonal and systemic urinary biomarkers in PD.

Author (Year)	Study Design/Population	Biomarker Class	Urinary Biomarker(s)	Analytical Method	Main Findings	Clinical Relevance
Gaikwad et al. (2011) [60]	Case–control (PD, HC)	Hormonal/Genotoxic	Estrogen-derived DNA adducts (4-OHE1(E2)-1-N3Ade; 4-OHE1(E2)-1-N7Gua)	LC–MS/MS	Estrogen-DNA adducts ↑ (PD)	Systemic oxidative/ genotoxic stress
Knezevic et al. (2023) [61]	Case–control (PD, HC)	Hormonal/Endocrine	Steroid hormones	LC–MS/MS	Steroid metabolism altered	Systemic endocrine changes
Luan et al. (2015a) [36]	Case–control (PD, HC)	Hormonal/Endocrine	Cortisol, 11-deoxycortisol, 21-deoxycortisol	GC–MS metabolomics	Cortisol-related metabolites ↑ (PD)	HPA axis dysregulation
Soares et al. (2019) [62]	Case–control (PD, HC)	Hormonal/Stress-related	Cortisol metabolites	Immunoassay	Altered cortisol profile (PD)	Stress response
Wang et al. (2023) [39]	Case–control (PD, HC)	Neurotransmitter-related	3-methoxytyramine, N-acetyl-L-tyrosine	LC–MS/MS metabolomics	Dopamine-related metabolites ↑ (PD)	Dopaminergic metabolism

PD, Parkinson’s disease; HC, healthy controls; HPA, hypothalamic–pituitary–adrenal; LC–MS/MS, liquid chromatography–tandem mass spectrometry; GC–MS, gas chromatography–mass spectrometry. Arrows indicate the direction of change in PD compared with controls (↑ increased).

## Data Availability

No new data were generated in this study. All data supporting the findings are derived from published literature and are included in the manuscript.

## Abbreviations

PD, Parkinson’s disease; CSF, cerebrospinal fluid; EV, extracellular vesicle; uEV, urinary extracellular vesicle; BBB, blood–brain barrier; iRBD, isolated rapid eye movement sleep behavior disorder; LRRK2, leucine-rich repeat kinase 2; GBA, glucocerebrosidase gene; GCase, glucocerebrosidase; BMP, bis(monoacylglycerol)phosphate; α-syn, alpha-synuclein; αSyn-SAA, alpha-synuclein seeding amplification assay; GWAS, genome-wide association study; SNP, single-nucleotide polymorphism; NMC, non-manifesting carrier; Rab, Rab GTPase; TCA, tricarboxylic acid cycle; ROS, reactive oxygen species; 8-OHdG, 8-hydroxy-2′-deoxyguanosine; HPA, hypothalamic–pituitary–adrenal; TMAO, trimethylamine N-oxide; KYN, kynurenine; KYNA, kynurenic acid; QUIN, quinolinic acid; LC–MS/MS, liquid chromatography–tandem mass spectrometry; GC–MS, gas chromatography–mass spectrometry; sFIDA, surface-based fluorescence intensity distribution analysis; ELISA, enzyme-linked immunosorbent assay; PRM, parallel reaction monitoring; WB, Western blot; UPDRS, Unified Parkinson’s Disease Rating Scale; MoCA, Montreal Cognitive Assessment; DALYs, disability-adjusted life years; WHO, World Health Organization; BAPC, Bayesian age–period–cohort; PRISMA, Preferred Reporting Items for Systematic Reviews and Meta-Analyses; SNAP23, synaptosome-associated protein 23; CALB1, calbindin 1; ICAM1, intercellular adhesion molecule 1; AHCY, adenosylhomocysteinase; STOM, stomatin; GM2A, GM2 activator protein.
